# Surface Acoustic Waves Enhance Neutrophil Killing of Bacteria

**DOI:** 10.1371/journal.pone.0068334

**Published:** 2013-08-06

**Authors:** John D. Loike, Anna Plitt, Komal Kothari, Jona Zumeris, Sadna Budhu, Kaitlyn Kavalus, Yonatan Ray, Harold Jacob

**Affiliations:** 1 Department of Physiology and Cellular Biophysics, Columbia University College of Physicians and Surgeons, New York, New York, United States of America; 2 NanoVibronix, Nesher, Israel; 3 Harvard University Medical School, Boston, Massachusetts, United States of America; 4 Department of Immunology, Sloan-Kettering Institute, New York, New York, United States of America; 5 Regeneron Pharmaceutical Corp, Tarrytown, New York, United States of America; Massachusetts General Hospital and Harvard Medical School, United States of America

## Abstract

Biofilms are structured communities of bacteria that play a major role in the pathogenicity of bacteria and are the leading cause of antibiotic resistant bacterial infections on indwelling catheters and medical prosthetic devices. Failure to resolve these biofilm infections may necessitate the surgical removal of the prosthetic device which can be debilitating and costly. Recent studies have shown that application of surface acoustic waves to catheter surfaces can reduce the incidence of infections by a mechanism that has not yet been clarified. We report here the effects of surface acoustic waves (SAW) on the capacity of human neutrophils to eradicate *S. epidermidis* bacteria in a planktonic state and within biofilms. Utilizing a novel fibrin gel system that mimics a tissue-like environment, we show that SAW, at an intensity of 0.3 mW/cm^2^, significantly enhances human neutrophil killing of *S. epidermidis* in a planktonic state and within biofilms by enhancing human neutrophil chemotaxis in response to chemoattractants. In addition, we show that the integrin CD18 plays a significant role in the killing enhancement observed in applying SAW. We propose from out data that this integrin may serve as mechanoreceptor for surface acoustic waves enhancing neutrophil chemotaxis and killing of bacteria.

## Introduction

Bacteria can exist in the body in a planktonic state, as single cell organisms, or as aggregates of microbes with a distinct architecture, called biofilms. The sessile communities of bacteria in biofilms, attached to a surface, are encased in a matrix composed primarily of polysaccharides, extracellular DNA, and protein and mediate both cell–surface and cell–cell interactions [1]. A major cause of infections of indwelling catheters and prosthetic devices is the formation of biofilms by *Staphylococcus epidermidis* bacteria [2]. Biofilms forming on the surface of catheter lines, prosthetic devices, pacemakers, contact lenses, heart valve replacements, artificial joints, and other surgical implants represent a major source of chronic wound pathogenesis [3], [4]. Despite numerous attempts at reducing infections due to biofilm formation on indwelling catheters, biofilms still cause over 80% of microbial infections [4], and often result in prolonged hospitalization and even morbidity [5].

Normal eradication of bacteria in infections requires the cytolytic activities of cells of the innate immune system such as neutrophils and macrophages. In addition, the human body often generates a fibrin matrix at the site of infection that prevents the bacteria from spreading to other sites to retard bacterial spreading to other areas of the body. Fibrin-containing sites are well-suited for neutrophils to phagocytose and kill the bacteria [6]. Yet, the development of a fibrin sheath resulting from biofilm formation represents a common cause of catheter dysfunction and failure [7].

Biofilms may be up to a 1,000-fold more resistant to antibiotics than planktonic bacteria [8]. The mechanisms for this resistance to antibiotics are not clear. It is possible that the presence of an exopolysaccharide “slime” matrix formed by bacteria within the biofilm retards the accessibility of antibiotics to the bacteria [8], [9]. Another reason may be related to the observation that bacteria embedded in biofilms grow at a slower rate than planktonic bacteria, making them more resistant to the anti-proliferative actions of antibiotics [10]. Bacteria within biofilms may also be less accessible to immune cells interfering with the capacity of immune cells to interact with bacteria within the biofilm [11].

Numerous studies have aimed at developing therapeutic approaches to eradicate the bacteria within biofilms, but none have been completely successful. Studies have showed that applying low energy surface acoustic waves (SAW) to catheters reduced biofilm formation on urinary catheters inserted into rabbits [8], [12]. These authors hypothesized that SAW would be effective on various surfaces regardless of the type of bacteria [8]. The exact mechanism by which the acoustic waves prevented biofilm formation remains to be characterized. In addition, it is not clear how SAW impedes biofilm formation. It may directly affect bacteria growth within biofilms or it may enhance the cytolytic capacity of critical immune cells, such as neutrophils.

Here, we have employed a fibrin gel system [13]–[16] to examine the effects of SAW on neutrophil-mediated killing of bacteria. We show that SAW enhances killing of *S. epidermidis,* maintained as planktonic bacteria or within biofilms, by human and by murine neutrophils. We also show that SAW enhances recruitment of neutrophils into the fibrin gels which is, in part, mediated by the neutrophil CD18 (β_2_) integrins. Inactivation of these integrins using EDTA or using neutrophils from mice deficient in CD18 integrins, dramatically reduced the biological properties of SAW on neutrophils. Our results provide insights into why administration of SAW reduces biofilm formation and how SAW may decrease the risk of infections in patients who have recently received a urinary catheter or other prosthetic devices.

## Materials and Methods

**Figure 1:**
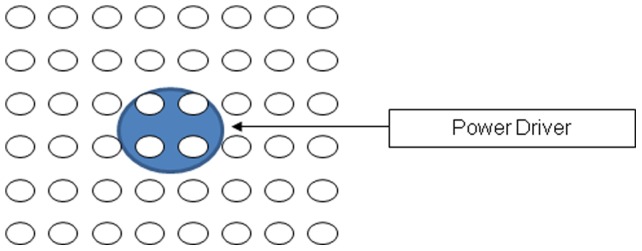
Schematic of 48-well plates connected to the piezo ceramic element. The element is attached to the bottom of the plate as depicted in the oval and is connected to an electronic driver powered by a 3.5 V battery.

Low energy surface acoustic waves (SAW) were delivered from a battery-powered driver (NanoVibronix Inc., Farmingdale, New York) that sends periodic electrical pulses to an actuator harboring a ceramic piezo element. The frequency of the vibrations generated on the piezo element is 100 kHz +10% and at on/off frequency of 30 Hz; the acoustic intensity was 0.4 Wcm^2^ and an amplitude of 2 mm. The driver is designed and calibrated for each cell culture plate (24, 48, and 96 well plate and see Fig. 1) to deliver 0.3 mW/ cm^2^ (ranging between 95–220 kHz) [8] for each type of cell culture plate. The SAW energy levels used in these experiments are used clinically in the UroShield and PainShield devices. (NanoVibronix Inc., Farmingdale, New York). Calibration was done by conducting pressure and intensity measurements with a Hydrophone needle probe method using the Precision Acoustics High Performance Hydrophone System: The accuracy of the measurements ±20%.

In order to ensure that the energy delivered by these devices did not affect temperature, we assessed the temperature in each well of the plates. We added PBS to each well of 24, 48 and 96 well plates connected to the SAW driver and placed the plates in a 37°C incubator for 2 hr. Every 15 mins the temperature of each well was recorded using an electronic thermometer. We observed no significant differences in temperature across the plates. In 24-transwell plates used to measure chemotaxis and bacterial killing, the temperature in both control and SAW applied wells remained at 37°C for six hrs (data not shown). Notwithstanding, no experiments were done in wells that were in direct contact with the ceramic piezo element.

### Mice

Breeding pairs of CD18-deficient C57Bl/6 (CD18−/−) [17] mice were generously provided by Dr. Claire Doerschuk (Rainbow Babies and Children Hospital, Cleveland, OH). All mice were housed and bred in a barrier facility at Columbia University. Mice 6–20 weeks of age were used according to protocols approved by Columbia University's Animal Care and Use Committee.

### Phagocytosis Assay

Human neutrophils were isolated from human blood using a two-layer Histopaque gradient as previously described [18]. Murine neutrophils were isolated from bone marrow as described [19]. Neutrophils (from human blood or from the bone marrow of mice) were re-suspended in phosphate buffered saline containing 5.5 mM glucose, 0.1% Human Serum Albumin (PBS-G-HSA) [18]. 10^6^ neutrophils from each preparation were added to each well of a 24-well plate containing sterilized 12 mm diameter glass coverslips containing 1 mL of RPMI medium supplemented with 2.5×10^−7^ M Lipopolysaccharide (LPS) and either 10% human serum (for human neutrophils) or 10% fetal bovine serum (for murine bone marrow neutrophils. LPS stimulated the cells to rapidly adhere to the glass coverslips within 30 mins. After cell adhesion at 37°C, 20 μL of a 1% (v/v) rabbit antibody (Hardy Diagnostics, Santa Maria, CA) coated sheep red blood cells (RBCs) [18] were added to each well. Following an additional 90 mins of incubation at 37°C in the presence or absence of SAW, the phagocytic index of the cells was determined as described [18]. Phagocytic Index (PI) is the average number of RBCs ingested per 100 neutrophils. To assess the total number of attached and ingested RBCs per 100 neutrophils, cells were washed only in PBS without lysing the extracellular RBCs.

### FACS Assay to assess phagocytosis of bacteria by neutrophils

Freshly isolated Human Neutrophils were re-suspended in PBS-G-HSA (pH = 7.35) [18]. *S. epidermidis* bacteria (10^9^ cfu/mL) were incubated with 1.5 μM BCECF-AM (fluorescent dye) for 15 minutes at 37°C. The bacteria were then washed three times in PBS to remove extracellular dye and then opsonized for 30 mins in 40% normal human serum (NHS). Fibrin gels were formed in 48-well plates by the addition of 5×10^5^ freshly isolated human neutrophils with either 10^7^/mL or 10^8^/mL BCECF-AM labeled bacteria, 1 mg/mL human fibrinogen, and 40% Human Serum, in 100 uL of PBS-G-HSA buffer containing 5 μL of thrombin (.02 units/μL). After 15 mins incubation at 37°C, 10 μL of PPACK (10^−7^ M) was added to each gel to terminate thrombin-induced fibrinogen polymerization. The gels were then incubated for 90 mins at 37°C and then lysed for 30 mins at 37°C in PBS containing 200 μL of trypsin (5 mg/mL) and cytochalasin D (20 μM). 700 μL of solution PBS containing 10% fetal bovine serum, and 20 μM cytochalasin D were added to each well and the neutrophils were recovered by centrifugation (10 mins at 400g at 4°C). The cells were washed in PBS-G-HSA and resuspended in PBS-G-HSA containing 50 units/ml of stapholysin (50 u/mL) and cytochalasin D (20 μM) and incubated for 15 mins at 37°C to lyse extracellular or non-ingested bacteria. The neutrophils were resuspended in 500 μL PBS-G-HSA buffer analyzed using a BD FACS Calibur, to assay the number of fluorescence-labeled bacteria ingested per neutrophil. The number of bacteria ingested per neutrophil was calculated using a standard curve generated by FACS analysis of 10^4^–10^6^ CFU of labeled bacteria.

### H_2_O_2_ and Chemotaxis Assays

Hydrogen peroxide generation by human neutrophils was performed on cells adherent to 96-well plates pre-coated with fibrinogen as described [18]. Two identical plates were prepared, one plate served as the control and the other received SAW via the ceramic element and driver for the duration of the experiment. Standard concentrations of H_2_O_2_ were measured for each experiment as described [20]. The generation of H_2_O_2_ was assessed fluorometrically every 30 mins for three hours using a Cytoflour II plate reader calibrated to excite at 485 nm and record emission at 540 nm. Control experiments revealed that SAW did not affect the fluorescent intensity when measuring standard solutions of H_2_O_2_ (data not shown). H_2_O_2_ production was measured in the presence or absence of SAW under the following conditions: a) wells containing no neutrophils, b) wells in which untreated neutrophils were added, c) wells in which neutrophils were added in the presence of PMA (10^−9^ M) or fMLP (10^−7^ M).

Chemotaxis was measured as described [16] using 8 micron pore cell culture inserts (Becton Dickinson, Franklin Lakes, NJ) added to 24 well tissue culture plates to create chemotaxis chambers. Chemotaxis of neutrophils into the bottom chamber was determined using a Coulter counter after a 6 hr incubation as described which represents a time point where the maximum number of cells have migrated into the lower chamber in response to a chemoattractant [16].

### Bacterial Killing in a Fibrin Gels and within Biofilm

Bacterial killing in fibrin gels were assayed as described [13]. At the end of the experiment, gels were lysed with 200 μL PBS containing trypsin (5 mg/mL) and cytochalasin D (20 μM) and the number of colonies formed in agar plates was counted 24 hr later as described as described [13]. *S. epidermidis* also were grown as biofilms on 6 mm diameter sterile silicone discs [Degania Silicone LTD –thickness 0.5 mm] in 24-well plate containing 1.5 mL of Tryptic Soy Broth (TSB). Approximately 10^5^ CFU *S. epidermidis* were added to each well containing the silicone discs and allowed to grow at 37°C for 7–10 days. At the end of the incubation, the bacteria-coated discs were washed three times in saline and the numbers of bacteria were counted as described below. Bacteria containing discs were opsonized with 10% Human Serum in PBS-G-HSA for 30–60 minutes at 37°C before the addition of the neutrophils. Each disc was then gently washed three times in saline, and transferred to new wells in a 24-well plate containing 1 ml of PBS-G-HSA. As indicated human neutrophils were added to discs in the presence or absence of SAW at 37°C in a humidified CO_2_ incubator. After 90 min, the discs were transferred into 1.3 mL of sterile water, vortexed and sonicated with 20 pulses for 30 secs. No bacteria remained on the discs following sonication as observed under light microscopy (data not shown). Following two serial 10-fold dilutions in sterile water, 50 μL of the sonicated solution was added to bacterial TSB agar plates and incubated at 37°C. The bacterial colonies that formed were counted after 48 hour [14], [15]. At these dilutions, control plates incubated in the absence of neutrophils usually contained approximately 2,000 colonies, or 10^7^ bacteria per disc.

All experiments using live animals were approved by Columbia University's IACUC (#AC-AAAB2859) and performed in strict accordance with ethical guidelines following the Guide for the Care and Use of Laboratory Animals of the National Institutes of Health. All animals were maintained in the animal center of Columbia University that included procedures and steps to ensure animal welfare. When necessary, mice were anesthetized using carbon dioxide, followed by dislocation of the neck in accordance with Columbia University institutional guide lines. All surgeries on the mice were performed under sodium pentobarbital anesthesia, and all efforts were made to minimize suffering. Work involving the isolation of human neutrophils was performed with the approval of Columbia University's IRB committee for this study. All volunteers provided a written informed consent, approved by Columbia University's IRB, to participate in this study.

## Results

### SAW enhances neutrophil killing of bacteria in fibrin gels

**Figure 2:**
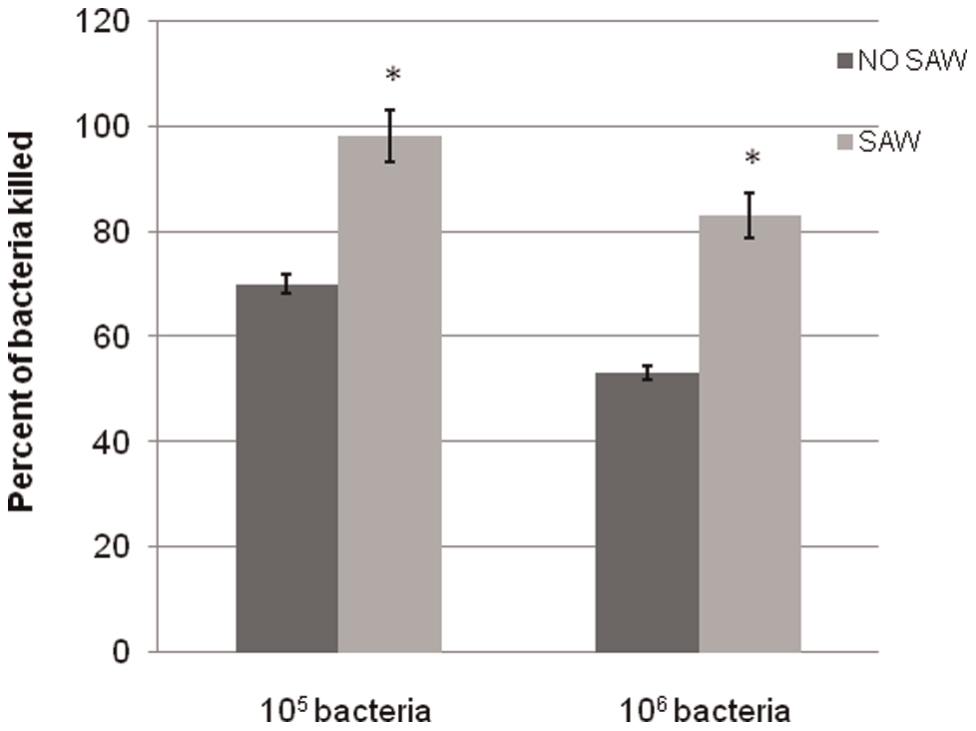
Killing of *S. epidermidis* bacteria in fibrin gels. *S. epidermidis* killing by 4×10^6^/mL human neutrophils was measured as described under Materials and Methods. The bacteria were opsonized with 10% human serum for 30 mins before incubating them with the neutrophils and the killing of the bacteria by the neutrophils was assessed after a 90 mins incubation period as described under Materials and Methods. Values are the percent killing ± SEM (n = 4). The difference between control and SAW treated cells are statistically significant at the p<0.001 level.

We first examined the effects of SAW on human neutrophil killing of planktonic *S. epidermidis* bacteria using a fibrin gel system [14], [15] that mimics a tissue-like environment (Fig. 2). The effects of SAW on bacterial killing by human neutrophils (4×10^6^ human neutrophils/mL) were examined in fibrin gels containing 10^5^/mL and 10^6^/mL S. epidermidis after a 90 mins incubation at 37°C as described in Materials and Methods. In the absence of SAW, human neutrophils killed about 70% of 10^5^ and 55% of 10^6^ of *S. epidermidis* in fibrin gels within 90 mins (Fig. 2). Applying SAW during the 90 incubation with neutrophils resulted in almost 100% neutrophil-mediated killing of 10^5^ CFU (p<0.001) and increased the percent of neutrophil-mediated bacteria killing from 55% to 82% (p<0.001) in fibrin gels containing 10^6^ bacteria (Fig. 2).

### SAW enhances neutrophil chemotaxis through fibrin gels

**Figure 3:**
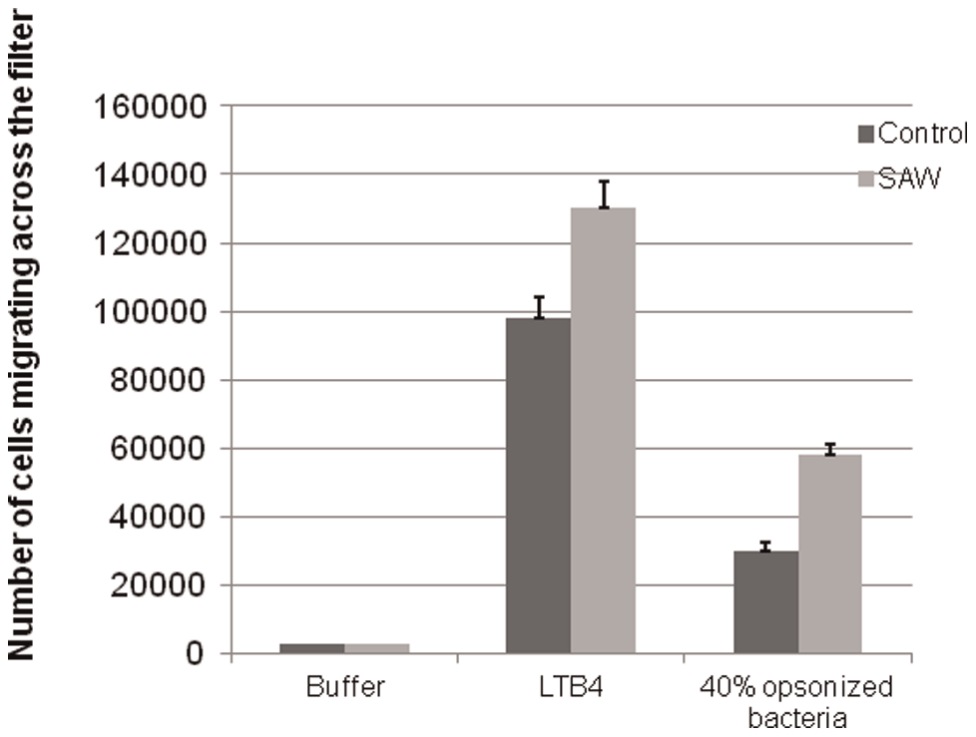
Neutrophil chemotaxis in response to LTB4 and opsonized bacteria. Neutrophil chemotaxis was measured as described [16]. Where indicated, the chambers were exposed to SAW for 6 hr immediately after the addition of the human neutrophils to the upper compartment of the transwell chemotaxis chambers as described under Materials and Method [16]. The bottom compartments contained 0.5 mL of either PBS-G-HSA, buffer alone, or buffer supplemented with 10^−7^ M LTB4 or 10^8^ CFU *S. epidermidis* opsonized with 40% human serum. Values represent the number of cells in the lower compartment ± SEM of n = 3 experiments performed in duplicates. *Difference between control and SAW treated cells is significant at p<0.05 level when either LTB4 or opsonized bacteria were added as chemoattractants to the lower compartment.

There are several mechanisms that could account for the observed increase in bactericidal activity of neutrophils in the presence of SAW. These include chemotaxis, phagocytosis, and the generation of hydrogen peroxide (H_2_O_2_). We therefore examined the effect of SAW on these neutrophil effector functions. Neutrophil chemotaxis was assessed using 8 micron pore cell culture inserts coated with a fibrin gel as previously described [21]. In the absence of any chemoattractants, few neutrophils (<50 cells) migrated from the upper chamber across fibrin gel into the lower chamber after a 6 hr incubation (Fig. 3). As previously reported [16], in the absence of SAW the chemoattractant leukotriene B4 (LTB4) (10^−7^ M) dramatically enhances neutrophil migration across fibrin gels such that approximately ∼10% of the total neutrophils added (100,000 cells) migrated after 6 hr into the lower chamber. However, when SAW was applied, chemotaxis in response to 10^−7^ M LTB4 significantly increased chemotaxis by almost 50% resulting in about 15% of the total neutrophils added (150,000 cells) migrating from the upper chamber into the lower chamber (Fig. 3). Control experiments showed that in the absence of LTB4 the application of SAW did not increase chemotaxis across fibrin gels (Fig. 3). To examine chemotaxis by bacterial generated chemoattractants we first pre-opsonized *S. epidermidis* (10^8^ CFU-) with 40% Human serum for 30 mins and placed them in the lower chamber of the cell inserts. In the absence of SAW, approximately 3% of the neutrophils (30,000 cells) migrated after 6 hr into the lower compartment in response to substances generated by opsonized bacteria. When SAW was applied during the 6 hour incubation period, neutrophil chemotaxis was enhanced by almost two fold in response to opsonized bacteria such that 55,000 neutrophils migrated into the lower compartment (Fig. 3).

### Enhanced chemotaxis by SAW requires β-2 (CD11/CD18) integrins

Previous work in our laboratory has demonstrated the critical role that CD18 integrins plays in mediating neutrophil killing of *S. epidermidis* opsonized with 40% human serum [13]–[15]. Clinically, patients deficient in CD18, exhibit leukocyte adhesion deficiency and suffer from life-threatening bacterial infections [22]. We therefore examined whether integrins have any role in the effects of SAW on three biological properties of neutrophils, bacterial killing, chemotaxis, and the production of hydrogen peroxide.

**Figure 4:**
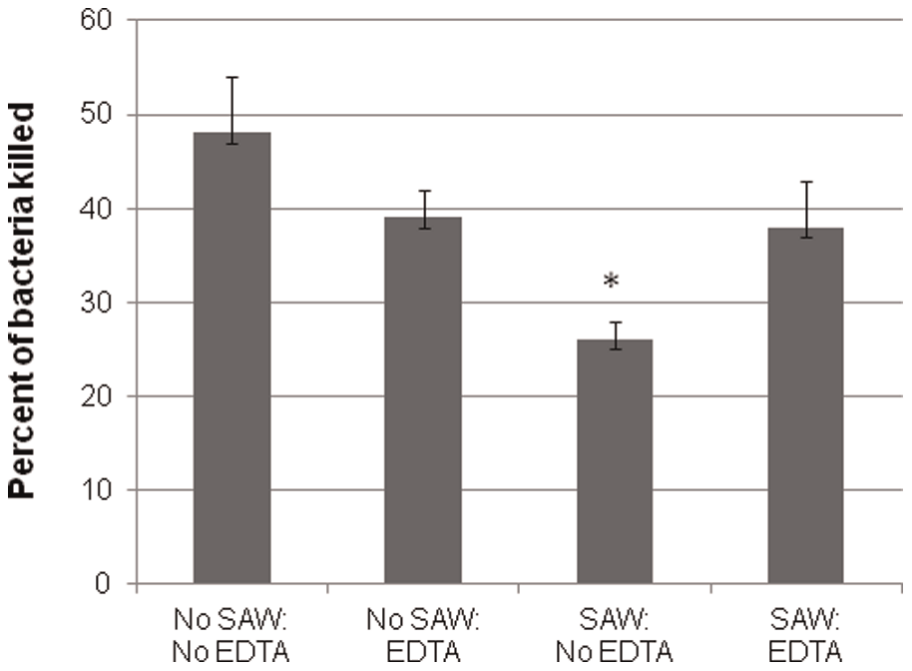
The effects of EDTA on neutrophil mediated killing of bacteria in fibrin gels. Bacterial killing was done by co-embedding human neutrophils (10^5^ cells/gel) with 10^5^ CFU *S. epidermidis* in the presence or absence of 5 mM EDTA and incubated at 37°C for 90 mins. The gels were then lysed and bacteria counted as described under Materials and Methods. Shown is the percent ± SEM of *S. epidermidis* killed by neutrophils in a representative experiment (n = 3).

The capacity of neutrophils to kill bacteria within fibrin gels does not, in fact, depend upon integrin activation (manuscript in preparation). The presence of 5 mM EDTA in the fibrin gel had no effect upon the percent of *S. epidermidis* killed by human neutrophils. (Fig. 4). As expected, applying SAW to these gels in the absence of EDTA increased the percent of bacteria killed within a 90 mins incubation period (Fig. 4). Yet, applying SAW in the presence of 5 mM EDTA completely abrogated the enhanced killing of bacteria observed in the absence of EDTA (Fig. 4).

We also examined the effects of EDTA and SAW on chemotaxis. In order to examine the role of CD18 integrins and the effects SAW on chemotaxis, we took advantage of our unpublished results that human neutrophils migrate from within fibrin gel into the lower compartment of a chemotaxis chamber in the presence of 5 mM EDTA. In contrast, adding EDTA to human neutrophils will block adhesion to the fibrin gels and prevent further migration (manuscript in preparation). By resuspending human neutrophils within a fibrin gel, we could observe chemotaxis even in the presence of EDTA. Therefore, we examined the migration of human neutrophils embedded in fibrin gels into the lower compartment of the chemotaxis chamber in the presence of 5 mM EDTA. The application of SAW did not enhance the migration of human neutrophils in response to LTB4 in the presence of 5 mM EDTA (Table 1). This is in contrast, to data in Fig. 3, which shows that applying SAW significantly enhanced human neutrophil migration in response to LTB4.

**Table 1:** SAW enhancement of neutrophil chemotaxis requires integrins.

	Chemotaxis^a^ in the presence of EDTA
Control	9.5±5%
SAW	9.6±4%

a 10^6^ human neutrophils were embedded in fibrin gels in the presence of 5 mM EDTA and were allowed to migrate from the gels into the lower compartment in response to 10^−7^ M LTB4. The number of cells that migrated was assessed as described under Materials and Methods. Shown is the percent ± SEM of 10^6^ neutrophils that migrated in response to LTB4 after 6 h. (N = 3).

**Figure 5:**
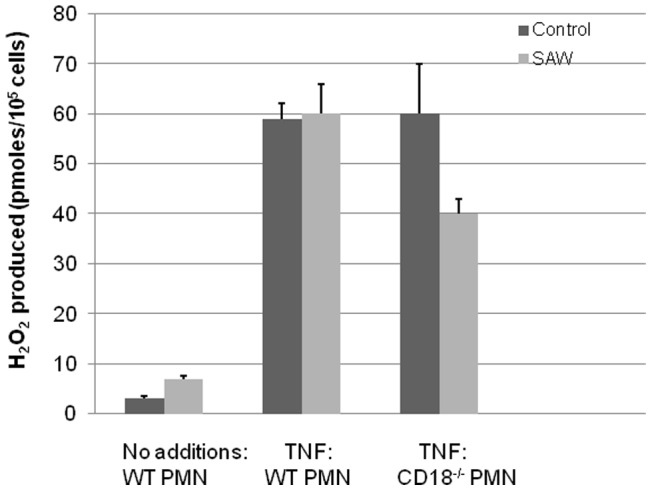
The effects of SAW on bone marrow derived neutrophils obtained from wild type and CD18^−/−^ deficient mice. 10^5^ Bone marrow derived neutrophils were prepared as described under Materials and Methods [30]. 10^5^ neutrophils [wild type (WT) and CD18 deficient (CD18^−/−^)] were incubated in the absence (Control) or in the presence of 100 ng/mL of TNFα. Values reported here represent the mean ± SEM of H_2_O_2_ produced by 10^5^ neutrophils incubated for 90 mins (n = 3). * = p<0.05.

We also examined the role of CD18 integrins and SAW on the generation of hydrogen peroxide by SAW. Wild type bone marrow derived neutrophils produced equal amounts H_2_O_2_ in the absence or presence of SAW in response to 100 ng/mL of the cytokine Tumor Necrosis Factor alpha (TNFα) [Fig. 5 and [20]]. We observed that applying SAW to CD18– deficient bone marrow derived neutrophils reduced their capacity to generate hydrogen peroxide within a fibrin gel in response to TNFα (Fig. 5). In contrast, CD18 deficient neutrophils produced equal amounts of H_2_O_2_ compared to wild type cells in the absence of SAW. It is important to point out that the application of SAW per se does not in itself stimulate H_2_O_2_ production (data not shown).

In summary, the data examining the effects of EDTA on humanneutrophil killing of bacteria and chemotaxis or using murine CD18 null bone marrow derived neutrophils to generate H_2_O_2_ revealed that beta 2 integrins play a significant role in generating a biological response to SAW.

### SAW does not enhance phagocytosis of *S. epidermidis* by human neutrophils

**Figure 6:**
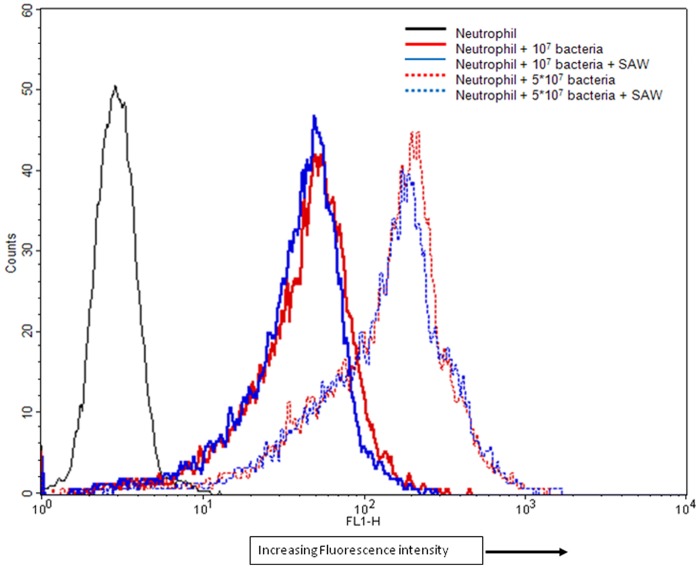
FACS analysis of neutrophil phagocytosis of BCECF-AM labeled *S. epidermidis*. 10^7^ and 5×10^7^ CFU *S. epidermidis* were co-embedded with human neutrophils in fibrin gels. After 90 mins the gels were lysed and the neutrophils were recovered and fluorescence intensity was analyzed using a BD FACS Calibur.

*In vivo*, neutrophils kill the bacteria via a variety of mechanisms including phagocytosis and/or release of extracellular hydrogen peroxide (H_2_O_2_). We assessed the effects of SAW on phagocytosis of opsonized BCEF-AM labeled *S. epidermidis* by human neutrophils within our fibrin gel system using FACS analysis (Fig. 6). 10^9^ *CFU of S. epidermidis* were labeled with 1.5 μM BCECF-AM, then opsonized with 40% human serum. 10^7^ to 5±10^7^ /mL of opsonized BCEF-AM labeled *S. epidermidis* were co-embedded with neutrophils (10^6^/ml) in 100 μL fibrin gels for 90 mins in the presence or absence of SAW. After 90 mins of incubation, the gels were lysed, the neutrophils were recovered and the extent of phagocytosis was analyzed by FACS. Neutrophils that phagocytosed the bacteria displayed an increase in the fluorescence from the ingested bacteria. As the number of bacteria is increased from 10^7^ to 5×10^7^ per ml, there was an increase in fluorescence intensity indicating that more bacteria were ingested by the neutrophils (Fig 6). However, no difference was observed in the intensity of fluorescence between control and SAW treated neutrophils.

### SAW enhances human neutrophil killing of S. epidermidis in biofilms

**Figure 7:**
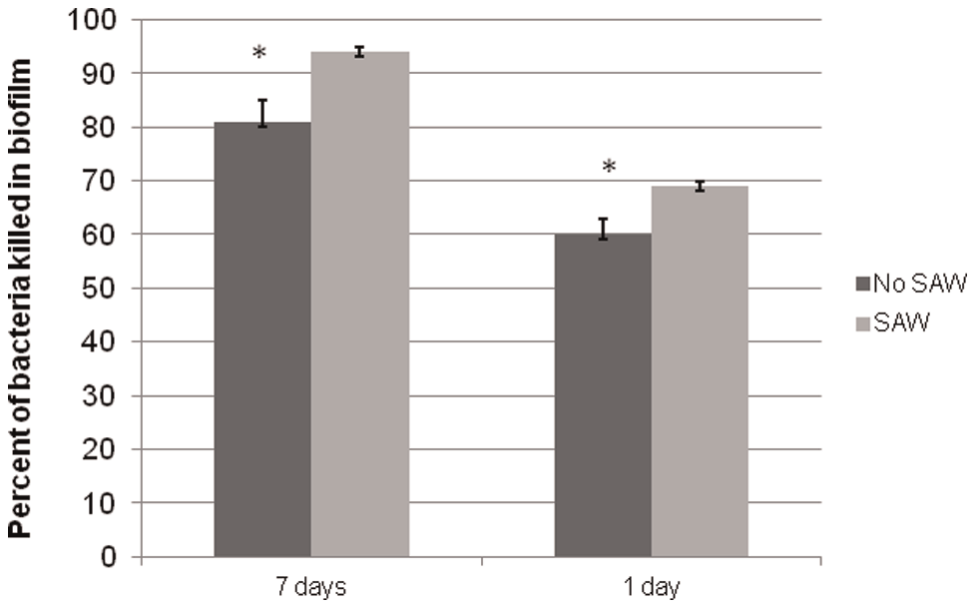
The Effect of SAW on Neutrophil Killing of Biofilms. Biofilms were cultured on silicone discs for 10 days. SAW was applied for either 7 consecutive days starting at day 3 to day 10 or for one day on day 10. During the 7 day applications, SAW was applied at each indicated time for 6 hr intervals (the battery life of the SAW device). For the one day application, SAW was applied for 90 mins on day 10. Freshly isolated human neutrophils were then added for 90 mins at 37°C on day 10 to biofilm-containing discs. As indicated, the plates were then further exposed to SAW during the incubation with neutrophils. At the end of the neutrophil co-incubation, the discs were removed, washed 3× in PBS and sonicated to release the bacteria from the discs as described under Materials and Methods. Bacterial growth was then assessed as described [13]. Samples were taken before the addition of the neutrophils and after the 90 mins co-incubation with neutrophils to assess viable bacteria (n = 2 done in triplicate). The percent of bacteria killed was calculated based on the control wells not incubated with neutrophils compared to those incubated with neutrophils. Student t-tests reveals significance at the p<0.05 level.

All of the above experiments were done on planktonic bacteria. We therefore measured the effects of SAW on neutrophil killing of *S. epidermidis* cultured as biofilms for 10 days, as described under Materials and Methods. As shown in Fig. 7, applying acoustic waves to the 10 day-old biofilms enhanced neutrophil bacterial killing within the biofilms by a small (11%) but significant (p<0.05) amount (Fig. 7).

We also examined the effects of longer exposure of SAW to biofilm containing discs. SAW was either applied each day for 6 hr (the life of the battery) for 3–10 days or for 90 mins after the biofilms developed for 10 days. Control experiments revealed that applying SAW to biofilm containing discs in the absence of neutrophils did not dislodge any bacteria. 10^7^ S. epidermidis/disc were recovered from biofilms grown in the presence or absence of SAW (data not shown). At the end of 10 days, neutrophils were added in the presence or absence of SAW. Biofilms exposed to repeated applications of SAW during the 3–10 days statistically enhanced neutrophil mediated killing at day 10 by 15%, while a single application of SAW to 10 day old biofilms enhanced killing by 10% (Fig. 7).

## Discussion

Bacteria often colonize the surfaces of catheters and prosthetic devices and grow as biofilm communities embedded in a gel-like polysaccharide matrix. Hazan et al., [8] showed that applying low-energy SAW prevented the formation of biofilms and interfered with the adhesion of planktonic microorganisms to the surfaces of indwelling devices. *In vivo* animal studies showed that applying SAW to Foley catheters inserted into rabbits dramatically reduced the incidence of bacteruria [8]. Recent reports [12], [23] show that SAW treatment of biofilms promotes the entry of antibiotics into the biofilm structure and dramatically increases bacterial killing. Further analysis suggested that the reduction of the incidence of biofilm formation was responsible for the reduced incidence of bacteruria in these animals [8].

We elected to examine the effects of SAW on neutrophils because these cells are primarily responsible for eradicating both planktonic bacteria and biofilms *in vivo*. Here, we show that applying SAW significantly increases the capacity of neutrophils to kill planktonic and biofilm bacteria using a fibrin gel model system that mimics the *in vivo* tissue environment [13]–[16], [20].

The precise mechanism by which SAW enhances neutrophil killing of bacteria is unknown so we designed experiments to examine the effects of SAW on specific neutrophil effector functions involved in their capacity to kill bacteria. Based on our data, we conclude that SAW does not promote increased phagocytosis or generate increases in hydrogen peroxide production by human neutrophils. However, we demonstrate that applying SAW significantly promotes neutrophil chemotaxis through fibrin gels in response to LTB4 and to chemoattractants generated in the medium by opsonized *S. epidermidis* (Fig. 3). This may relate to a recent report [1] showing that *S. aureus* biofilms actively attenuate traditional antibacterial immune responses, by significantly reducing cytokine/chemokine production associated with biofilm-infected tissues. At this time, it is unclear precisely how a SAW-mediated increase in chemotaxis leads to increased bacterial killing. One could speculate that the enhanced killing is due to increased killing of extracellular bacteria released from the biofilm. If this were the case then we would expect to observe an increase in hydrogen peroxide production. But, this was not possible to assess since hydrogen peroxide production is difficult to measure within fibrin gels. Thus, we measured hydrogen peroxide in neutrophils that adhered to fibrin-coated surfaces and found no effect of SAW on this process. For planktonic bacteria there are several reports that both IgG and C3b (and C3bi) opsonization enhances bacterial killing [24], [25]. In contrast, other studies [11] present data that the antibody opsonization of biofilms contributed little to the binding of, and the recognition by, neutrophils but lead to increased production of oxygen radicals. Experiments are underway to attempt to develop a better assay to assess extracellular hydrogen peroxide within fibrin gels.

Li et al., [13]–[15] showed that the neutrophil concentration, and not the ratio of effector to target cells, regulates bacterial killing by neutrophils in fibrin gels and in tissues. Thus, any process that enhances the body's capacity to reach or exceed the critical neutrophil concentration at the sites of infection should enhance bacterial killing and resolution of the infection. From our data, we propose that application of SAW enhances neutrophil chemotaxis to the sites of infection, which in turn decreases the time it takes to achieve the critical neutrophil concentration at these sites. Neutrophils may not be the only cell type whose chemotactic response is affected by SAW. We have reported that SAW also enhances T- cell chemotaxis in fibrin gels in response to IL-10 and Rantes.[26] Other studies (unpublished data- Dr. Li, Miami University) show that administration of SAW statistically increases cell migration of Human Keratinocytes (NIK) using a wound healing scratch assay *in vitro* model [27] and stimulates murine bone formation [28].

There are several potential mechanisms why SAW enhances neutrophil chemotaxis through fibrin gels. SAW may alter the gel structure by reducing internal effective friction forces between the cells and the extracellular matrix as they vibrate at different velocities in response to the same frequency. This view is consistent with ongoing studies that show that applying SAW to biofilms reduces the concentration of Gentamycin required to eradicate the bacteria and increases entry of antibiotics into biofilms [12]. Similarly, clinical studies show that surface acoustic vibrations decrease the effective friction forces of an NG tube surface on the nasal and pharyngeal tissues of patients [29].

### Role of Integrin CD18 in SAW

Our data uncovered the critical role that leukocyte integrins, and in particular CD18, play in the effects of SAW. Neutrophil chemotaxis through fibrin gels is sensitive to EDTA and *S. epidermidis* killing by human and murine neutrophils requires CD18 (manuscript in preparation). Furthermore, CD11/b/CD18 plays an important role in bacterial killing [13], [15], [16]. Our results (Fig. 4 and Table 1) show that EDTA- and TNFα- stimulated CD18-deficient murine neutrophils do not show enhanced bactericidal activity in response to SAW. These observations led us to propose that CD18 integrins may be sensitive to low energy ultrasound (SAW) and may activate these receptors. This, to our knowledge, is the first report of a cellular receptor whose function is impacted by SAW. Previous reports have shown that ultrasound induces mRNA expression of various chemokines including MCP-1, MIP-1b, MIP-2, and RANKL, in osteoblasts which are cells derived from bone marrow leukocytes [30]. We propose from out data that integrins such as CD18 may function as mechanoreceptors.

In summary, we show that surface acoustic waves significantly increase killing of opsonized *S. epidermidis* in fibrin gels by enhancing neutrophil chemotaxis. We propose that the SAW- mediated increase in killing bacteria in biofilms is due to the same mechanism.
